# Supplementary material for: High Use of Dietary Supplements and Low Adherence to the Mediterranean Diet Among Italian Adolescents: Results from the EduALI Project
Source: Nutrients. 2025 Jul 3;17(13):2213. doi: 10.3390/nu17132213 (PMC12251649; doi:10.3390/nu17132213)
Supplement: Supplementary file 1 [file nutrients-17-02213-s001.zip › nutrients-3711835-supplementary.pdf]

Come definire il tuo ID	Prime 2 lettere del nome	Prime 2 lettere del cognome	Giorno di nascita	ID completo
Es. Mario Rossi nato il 12 maggio	MA	RO	1205	MARO1205

Il tuo ID: __ __ __ __ __ __ __ __

SCHEDA ANAMNESTICA

Data ....

Nome della scuola: ....

☐ Maschio ☐ Femmina

Quanti anni hai: ....

Con quante persone vivi a casa: ....

Hai fratelli e/o sorelle? ☐SI ☐NO Se sì, quanti: ......…………

Quanto sei alto/a: ....

Quanto pesi: ....

ATTIVITÁ FISICA: Pratichi regolarmente uno sport? ☐SI ☐NO

Se sì, che tipo di attività fisica svolgi?

☐ Passeggiata all'aria aperta
☐ Corsa
☐ Calcio/calcetto
☐ Piscina
☐ Bicicletta
☐ Palestra
☐ Altro:..

Con che frequenza svolgi l'attività fisica?

☐Tutti i giorni; ☐Alcuni giorni della settimana (numero........)

DIETA ATTUALE

☐ Onnivora
☐ Vegetariana
☐ Vegana
☐ Senza glutine
☐ Senza lattosio
☐ Altro..................

Come definire il tuo ID	Prime 2 lettere del nome	Prime 2 lettere del cognome	Giorno di nascita	ID completo
Es. Mario Rossi nato il 12 maggio	MA	RO	1205	MARO1205

Il tuo ID: __ __ __ __ __ __ __ __

Qual è il consumo dei seguenti gruppi alimentari?

Questionario di aderenza alla dieta Mediterranea

**FRUTTA**<br>*1 porzione: 150 g (esempio: 1 mela, pera, arancia; 3 prugne, mandarini)*	<1 porzione/die ☐	1-2 porzioni/die ☐	>2 porzioni/die ☐
**VERDURA**<br>*1 porzione: 100 g (esempio: 1 piatto di insalata; 2 pomodori; ½ vaschetta di verdura cotta)*	<1 porzione/die ☐	1-2,5 porzioni/die ☐	>2,5 porzioni/die ☐
**LEGUMI**<br>*1 porzione: 70 g (esempio: ½ scatoletta di fagioli o ceci o lenticchie o piselli)*	<1 porzione/sett. ☐	1-2 porzioni/sett. ☐	>2 porzioni/sett. ☐
**CEREALI** (pane, pasta, biscotti etc.)<br>*1 porzione: 130 g (Esempi: 1 porzione pasta: 80 g; 4 biscotti frollini: 50 g)*	<1 porzione/die ☐	1-1,5 porzioni/die ☐	>1,5 porzioni/die ☐
**PESCE** (eccetto molluschi e crostacei)<br>*1 porzione: 100 g*	<1 porzione/sett. ☐	1-2,5 porzioni/sett. ☐	>2,5 porzioni/sett. ☐
**CARNE E SALUMI**<br>*1 porzione: 80 g (Esempi: 1 porzione carne: 100 g 1 porzione salumi: 50 g (esempio: ½ vaschetta prosciutto)*	<1 porzione/die ☐	1-1,5 porzioni/die ☐	>1,5 porzioni/die ☐
**LATTE E LATTICINI**<br>*1 porzione: 180 g (Esempi: 1 tazza di latte: 150 g; 1 yogurt: 125)*	<1 porzione/die ☐	1-1,5 porzioni/die ☐	>1,5 porzioni/die ☐
**ALCOL**<br>*1 U.A. = 1 bicchiere di vino; 1 lattina birra*	<1 U.A./die ☐	1-2 U.A./die ☐	>2 U.A./die ☐
**OLIO D'OLIVA**	Occasionalmente ☐	Frequentemente ☐	Regolarmente ☐

Totale: ☐

Adattato da Sofi et al., Public Health Nutr 2014

Come definire il tuo ID	Prime 2 lettere del nome	Prime 2 lettere del cognome	Giorno di nascita	ID completo
Es. Mario Rossi nato il 12 maggio	MA	RO	1205	MARO1205

Il tuo ID: __ __ __ __ __ __ __ __

QUESTIONARIO ALIMENTARE

A. CARNE, PESCE E UOVA

1. Mangi **CARNE ROSSA**? Si □ No, mai □

   Quanto? Porz. piccola (80 g) □ Porz. media (120 g) □ Porz. grande (200 g) □

   Quante volte alla settimana? .... *Meno di una volta a settimana* □

2. Mangi **CARNE BIANCA**? Si □ No, mai □

   Quanto? Porz. piccola (80 g) □ Porz. media (120 g) □ Porz. grande (160 g) □

   Quante volte alla settimana? .... *Meno di una volta a settimana* □

3. Mangi **AFFETTATI**? Si □ No, mai □

   Quanto? Porz. piccola (20 g) □ Porz. media (40 g) □ Porz. grande (80 g) □

   Quante volte alla settimana? .... *Meno di una volta a settimana* □

4. Mangi **PESCE**? Si □ No, mai □

   Quanto? Porz. piccola (100 g) □ Porz. media (150 g) □ Porz. grande (200 g) □

   Quante volte alla settimana? .... *Meno di una volta a settimana* □

5. Mangi **UOVA?** Si □ No, mai □

   Quante volte alla settimana? .... *Meno di una volta a settimana* □

B. LATTE E LATTICINI

6. Bevi **LATTE**? Si □ No, mai □

   Che tipo? Intero □ Parzialmente scremato □ Scremato □

   Quanto? Porz. piccola (120 ml) □ Porz. media (200 ml) □ Porz. grande (300 ml) □

   Quante volte alla settimana? .... *Meno di una volta a settimana* □

7. Mangi **YOGURT**? Si □ No, mai □

Quanto? Porz. piccola (125 ml) □ Porz. media (150 ml) □ Porz. grande (180 ml) □

Quante volte alla settimana? .... *Meno di una volta a settimana* □

8. Mangi **FORMAGGI?** Si □ No, mai □

Quante volte alla settimana? .... *Meno di una volta a settimana* □

FORMAGGI STAGIONATI *(parmigiano, pecorino, ecc.)* Si □ No, mai □

Quanto? Porz. piccola (30 g) □ Porz. media (50 g) □ Porz. grande (70 g) □

Quante volte alla settimana? .... *Meno di una volta a settimana* □

FORMAGGI FRESCHI *(mozzarella, stracchino, ecc.)* Si □ No, mai □

Quanto? Porz. piccola (50 g) □ Porz. media (70 g) □ Porz. grande (100 g) □

Quante volte alla settimana? .... *Meno di una volta a settimana* □

C. CEREALI

9. Mangi **PASTA o RISO**? Si □ No, mai □

Quanto? Porz. piccola (40-50 g) □ Porz. media (60-80 g) □ Porz. grande (100-120 g) □

Quante volte alla settimana? .... *Meno di una volta a settimana* □

10. Mangi **PANE**? Si □ No, mai □

Quanto? Porz. piccola (30 g) □ Porz. media (50 g) □ Porz. grande (100 g) □

Quante volte alla settimana? .... *Meno di una volta a settimana* □

11. Mangi **PRODOTTI DA FORNO**? Si □ No, mai □

Quanto? Porz. piccola (15 g) □ Porz. media (30 g) □ Porz. grande (45 g) □

Quante volte alla settimana? .... *Meno di una volta a settimana* □

12. Mangi **PATATE**? Si □ No, mai □

Quanto? Porz. piccola (100 g) □ Porz. media (150 g) □ Porz. grande (200 g) □

Quante volte alla settimana? .... *Meno di una volta a settimana* □

13. Mangi **PIZZA**? Si □ No, mai □

Quanto? Porz. piccola (100 g) □ Porz. media (150 g) □ Porz. grande (300 g) □

Quante volte alla settimana? .... *Meno di una volta a settimana* □

D. VERDURA, LEGUMI e FRUTTA

14. Mangi **VERDURA**? Si □ No, mai □

Quanto? Porz. piccola (100 g) □ Porz. media (150 g) □ Porz. grande (200 g) □

Quante volte alla settimana? .... *Meno di una volta a settimana* □

15. Mangi **LEGUMI** (fagioli, piselli, ecc.)? Si □ No, mai □

Quanto? *SECCHI:* Porz. piccola (30 g) □ Porz. media (50 g) □ Porz. grande (70 g) □

COTTI: Porz. piccola (75 g) □ Porz. media (125 g) □ Porz. grande (175 g) □

Quante volte alla settimana? .... *Meno di una volta a settimana* □

16. Mangi **FRUTTA FRESCA**? Si □ No, mai □

Quanto? Porz. piccola (100 g) □ Porz. media (150 g) □ Porz. grande (200 g) □

Quante volte alla settimana? .... *Meno di una volta a settimana* □

E. CONDIMENTI

17. Mangi **OLIO D'OLIVA**? Si □ No, mai □

Quanto? 1 cucchiaino (5 g) □ 2 cucchiaini (10 g) □ 3 cucchiaini (15 g) □

Quante volte alla settimana? .... *Meno di una volta a settimana* □

18. Mangi **OLIO DI SEMI**? Si □ No, mai □

Quanto? 1 cucchiaino (5 g) □ 2 cucchiaini (10 g) □ 3 cucchiaini (15 g) □

Quante volte alla settimana? .... *Meno di una volta a settimana* □

19. Mangi **BURRO**? Si □ No, mai □

Quanto? Porz. piccola (5 g) □ Porz. media (10 g) □ Porz. grande (15 g) □

Quante volte alla settimana? .... *Meno di una volta a settimana* □

20. Mangi **MARGARINA**? Si □ No, mai □

Quanto? Porz. piccola (5 g) □ Porz. media (10 g) □ Porz. grande (15 g) □

Quante volte alla settimana? .... *Meno di una volta a settimana* □

F. BIBITE

21. Bevi **CAFFÈ**? Si □ No, mai □

Quante volte al giorno? ....

22. Bevi **BEVANDE ALCOLICHE**? Si □ No, mai □

Quanto? *Vino:*	0.15 l □	0.20 l □	0.25 l □	
Birra:	0.23 l □	0.33 l □	0.45 l □	0.66 l □
Superalcolici:	0.03 l □	0.13 l □	0.20 l □	0.30 l □

Quante volte alla settimana? .... *Meno di una volta a settimana* □

23. Bevi **BIBITE** (coca-cola, succhi di frutta, ecc.)? Si □ No, mai □

Quante volte a settimana? ....

G. ALTRO

24. Mangi **DOLCI**? Si □ No, mai □

Quanto? Porz. piccola (30 g) □ Porz. media (50 g) □ Porz. grande (70 g) □

Quante volte alla settimana? .... *Meno di una volta a settimana* □

25. Mangi **CIBI FRITTI**? Si □ No, mai □

Quante volte alla settimana? .... *Meno di una volta a settimana* □

26. Mangi **FAST FOOD** (hamburger, hot-dog, ecc.)? Si □ No, mai □

Quanto? Porz. media (120 g) □ Porz. grande (210 g) □

Quante volte alla settimana? .... *Meno di una volta a settimana* □

Come definire il tuo ID	Prime 2 lettere del nome	Prime 2 lettere del cognome	Giorno di nascita	ID completo
Es. Mario Rossi nato il 12 maggio	MA	RO	1205	MARO1205

Il tuo ID: __ __ __ __ __ __ __ __

QUESTIONARIO SULL'ATTIVITÀ FISICA QUOTIDIANA (IPAQ)

Questo questionario misura il tipo e la quantità di attività fisica che lei fa normalmente. Le domande si riferiscono all'attività svolta negli ultimi 7 giorni **al lavoro**, **per spostarsi** da un posto all'altro e **nel tempo libero.** Nel rispondere alle domande, tenga conto solo di quelle attività che l'hanno impegnata per **almeno 10 minuti** consecutivi.

- Per attività fisica **MODERATA** si intende un'attività che richiede uno sforzo fisico moderato e che la costringe a **respirare con un ritmo solo moderatamente più elevato del normale** (durante tale attività non riuscirebbe a cantare ma le sarebbe ancora possibile parlare).
- Per attività fisica **INTENSA** si intende un'attività che richiede uno sforzo fisico elevato e che la costringe a **respirare con un ritmo molto più elevato del normale** (durante tale attività sisuda e non si riesce a parlare).

Attività intense

1a Negli ultimi 7 giorni, per quanti giorni ha compiuto attività fisiche **INTENSE**, come ad esempio sollevamento di pesi, lavori pesanti in giardino, attività aerobiche come corse o giri in bicicletta a velocità sostenuta?

________giorni alla settimana

Nemmeno uno → (vada alla domanda **2a**)

1b Quanto tempo in totale, normalmente, lei ha trascorso compiendo attività fisiche **INTENSE** in **uno** di questi giorni?

________minuti

Attività moderate

2a Negli ultimi 7 giorni, per quanti giorni ha compiuto attività fisiche **MODERATE**, come ad esempio trasporto di pesi leggeri, giri in bicicletta ad una velocità regolare, attività in palestra, lavoro in giardino, lavoro fisico prolungato in casa? Non consideri le camminate

________giorni alla settimana

Nemmeno uno → (vada alla domanda 3a)

2b Quanto tempo in totale, normalmente, lei ha trascorso compiendo attività fisiche **MODERATE** in **uno** di questi giorni?

________minuti

Cammino

3a Negli ultimi 7 giorni, per quanti giorni ha camminato per **almeno 10 minuti**?
(Consideri le camminate compiute al lavoro e a casa, quelle per spostarsi da un posto ad un altro ed ogni altra camminata che le è capitato di fare per piacere, esercizio o sport)

________giorni alla settimana

Nemmeno uno → (vada alla domanda **4a**)

3b Per quanto tempo in totale, normalmente, lei ha camminato in **uno** di questi giorni?

________minuti

3c A che passo ha camminato prevalentemente?

passo **INTENSO**, che l'ha fatta respirare ad un ritmo molto più elevato del normale
passo **MODERATO**, che l'ha fatta respirare ad un ritmo solo moderatamente più elevato del normale
passo **LENTO**, senza alcun cambiamento nel suo ritmo di respiro

Attività da seduto

4a Negli ultimi 7 giorni, quanto tempo in totale lei ha trascorso rimanendo seduto, durante **un giorno** lavorativo?
(includa attività svolte al lavoro, a casa, mentre si recava al lavoro e durante il tempo libero: es. ad una scrivania, a tavola, mentre stava visitando degli amici, alla TV,leggendo)

________minuti

4b Negli ultimi 7 giorni, quanto tempo in totale ha trascorso rimanendo seduto, durante **un giorno** del fine settimana?

________minuti

Come definire il tuo ID	Prime 2 lettere del nome	Prime 2 lettere del cognome	Giorno di nascita	ID completo
Es. Mario Rossi nato il 12 maggio	MA	RO	1205	MARO1205

Il tuo ID: __ __ __ __ __ __ __ __

QUESTIONARIO SULL'UTILIZZO DI INTEGRATORI ALIMENTARI

Per ogni domanda, segna una X accanto alla risposta scelta.

Anche se non hai mai utilizzato integratori alimentari, rispondi a tutte le domande e, se vuoi, puoi aggiungere commenti per approfondire la tua risposta.

1. Nell'ultimo anno hai utilizzato o assunto vitamine, minerali, prodotti erboristici o altri integratori alimentari?

   ____ SÌ
   ____ NO
   ____ NON SONO SICURO/A

   Se non sai cosa sono gli integratori alimentari, ecco alcuni esempi e marca con una crocetta quelli che utilizzi:

	**Vitamine**		**Erbe o prodotti botanici**		**Sali minerali**		**Altri integratori**
	Multivitaminico		Echinacea		Calcio		Amminoacidi e/o proteine
	Vitamina E		Aglio		Cromo		Creatina
	Vitamina C		Ginkgo		Ferro		Bevande sportive
	Vitamina A		Ginseng		Magnesio		Coenzima Q10
	Betacaroteni		Kava		Potassio		Olio di pesce
	Vitamine Gruppo C		Erba di San Giovanni - iperico		Selenio		Glucosamina
	Acido folico		Estratto di semi d'uva		Zinco		Melatonina
	Niacina		Saw Palmetto		Combinazione di più sali minerali		Omega 3
	Vitamina D						Acido alfa lipoico
							Acetil-L-carnitina

2. Pensi che l'assunzione di vitamine o di altri integratori, come sali minerali ed erbe, sia essenziale per la sua salute?

    ____ SÌ, È ESSENZIALE
    ____ NO, NON È ESSENZIALE
    ____ NON LO SO

    Commento:

3. Il medico ti ha consigliato di assumere vitamine, sali minerali o altri integratori specifici per la tua salute?

    ____ SÌ
    ____ NO
    ____ NON LO SO

Se sì, elencare quali:

Per ciascuna di queste affermazioni, indica se **sei d'accordo**, **neutrale**, **in disaccordo** o **se non lo sai**.

4. La quantità di sali minerali, vitamine e altre sostanze che ottengo dagli alimenti è sufficiente per le mie esigenze di salute.

    ____ SONO D'ACCORDO
    ____ MI SENTO NEUTRALE A RIGUARDO
    ____ SONO IN DISACCORDO
    ____ NON LO SO

    Commento:

5. Sono sicuro/a di capire quali vitamine, sali minerali, prodotti botanici e altri integratori sono adatti a me.

    ____ SONO D'ACCORDO
    ____ MI SENTO NEUTRALE A RIGUARDO
    ____ SONO IN DISACCORDO
    ____ NON LO SO

    Commento:

6. Le etichette degli integratori alimentari mi aiutano a capire se è l'integratore giusto per me.

    ____ SONO D'ACCORDO
    ____ MI SENTO NEUTRALE A RIGUARDO
    ____ SONO IN DISACCORDO
    ____ NON LO SO

    Commento:

7. Quali delle seguenti fonti ti danno informazioni sugli integratori alimentari più adatti a te? (Puoi scegliere più opzioni)

    ____ LIBRI
    ____ ETICHETTE DEGLI INTEGRATORI ALIMENTARI
    ____ FAMIGLIA
    ____ AMICI
    ____ RIVENDITORE DI ALIMENTI
    ____ RIVISTE/GIORNALI/QUOTIDIANI
    ____ IL MIO FARMACISTA
    ____ IL MIO MEDICO
    ____ INTERNET
    ____ ALTRE FONTI — Per favore, elenca quali:

    Commento:

8. Di quale delle seguenti fonti che ti danno informazioni sugli integratori alimentari ti fidi di più? (Si prega di segnare non più di 2 opzioni):
    ____ LIBRI
    ____ ETICHETTE DEGLI INTEGRATORI ALIMENTARI
    ____ FAMIGLIA
    ____ AMICI
    ____ RIVENDITORE DI ALIMENTI SALUTARI
    ____ RIVISTE/GIORNALI/QUOTIDIANI
    ____ IL MIO FARMACISTA
    ____ IL MIO MEDICO
    ____ INTERNET
    ____ ALTRE FONTI — Per favore, elenca quali:

    Commento:

    Il motivo principale per <u>cui assumo o non assumo</u> integratori è:

Riportaci gli integratori alimentari che usi:

Compila una tabella informativa (che trovi nelle schede seguenti) su vitamine, sali minerali, prodotti erboristici e altri integratori alimentari che assumi regolarmente. Utilizza le informazioni riportate sulle confezioni dei prodotti.

- √ Scrivi il nome completo dell'integratore, compreso il nome della marca e del produttore. Inserisci il maggior numero di informazioni possibili.
- √ Nei giorni in cui hai assunto il prodotto, quanto ne hai preso in un solo giorno?
- √ Da quanto tempo stai assumendo questo prodotto?
- √ Per quale motivo prendi questo prodotto? Altri commenti sul prodotto?

<table>
<tr><th colspan="6">Integratore n°1</th></tr>
<tr><td>Nome dell'integratore</td><td colspan="5"></td></tr>
<tr><td>Marca / produttore dell'integratore</td><td colspan="5"></td></tr>
<tr><td>Forma del prodotto</td><td>Capsula
Pacchetto/confezione
Gomma da masticare
Altra forma
(specificare:………………..)</td><td>Compressa
Liquido
Granulato</td><td>Pillole
Polvere</td><td colspan="2">Water
Gel</td></tr>
<tr><td>Quantità al giorno (includere la quantità di ogni dose)</td><td colspan="5"></td></tr>
<tr><td>Da quanto tempo stai assumendo questo prodotto</td><td>Giorni</td><td>Settimane</td><td>Mesi</td><td>Anni</td><td>Non lo so</td></tr>
<tr><td>Perché prendi l'integratore ed eventuali commenti</td><td colspan="5"></td></tr>
<tr><th colspan="6">Integratore n°2</th></tr>
<tr><td>Nome dell'integratore</td><td colspan="5"></td></tr>
<tr><td>Marca / produttore dell'integratore</td><td colspan="5"></td></tr>
</table>

Forma del prodotto	Capsula<br>Pacchetto/confezione<br>Gomma da masticare<br>Altra forma<br>(specificare:………………..)		Compressa<br>Liquido<br>Granulato	Pillole<br>Polvere	Water<br>Gel
Quantità al giorno (includere la quantità di ogni dose)					
Da quanto tempo stai assumendo questo prodotto	Giorni	Settimane	Mesi	Anni	Non lo so
Perché prendi l'integratore ed eventuali commenti					
**Integratore n°3**					
Nome dell'integratore					
Marca / produttore dell'integratore					
Forma del prodotto	Capsula<br>Pacchetto/confezione<br>Gomma da masticare<br>Altra forma<br>(specificare:………………..)		Compressa<br>Liquido<br>Granulato	Pillole<br>Polvere	Water<br>Gel
Quantità al giorno (includere la quantità di ogni dose)					
Da quanto tempo stai assumendo questo prodotto	Giorni	Settimane	Mesi	Anni	Non lo so
Perché prendi l'integratore ed eventuali commenti					
